# Nursing Caring Behaviors in Slovenian Emergency Departments Under High Workload Conditions: A Cross-Sectional Comparative Study of Patient and Nurse Perspectives

**DOI:** 10.3390/healthcare14172872

**Published:** 2026-09-07

**Authors:** Urška Fekonja, Matej Strnad, Zvonka Fekonja

**Affiliations:** 1 Emergency Department, University Medical Centre Maribor, 2000 Maribor, Slovenia; strnad.matej78@gmail.com (M.S.); zvonka.fekonja@um.si (Z.F.); 2 Faculty of Health Sciences, University of Maribor, 2000 Maribor, Slovenia; 3 Faculty of Medicine, University of Maribor, 2000 Maribor, Slovenia; 4 Prehospital Unit, Community Healthcare Center Maribor, 2000 Maribor, Slovenia

**Keywords:** caring behaviors, nurse, emergency department

## Abstract

**Highlights:**

**What are the main findings?**

- Nurses and patients in Slovenian emergency departments reported generally high levels of caring behaviors, with the highest ratings related to professional knowledge, technical competence, and patient safety.
- Patients consistently rated communication, responsiveness, and time availability lower than nurses, while longer waiting times and treatment duration were associated with lower perceptions of caring behaviors.

**What are the implications of the main findings?**

- Organizational factors such as workload, waiting times, and staffing conditions may be associated with how caring behaviors are experienced in emergency departments.
- Improving communication skills, responsiveness to patients’ needs, and workflow organization may strengthen patient-centered care and support better patient experiences in emergency settings.

**Abstract:**

**Background/Objectives**: Caring behaviors are key components of nursing practice and contribute to patient outcomes and satisfaction. In emergency departments (EDs), where care is delivered under time pressure, caring behaviors may be shaped by organizational constraints. Patients and nurses may perceive caring behaviors differently. This study aimed to translate, culturally adapt, and evaluate the psychometric properties of the CBI-24 and CBI-24-P for use in Slovenian EDs, assess the level of caring behaviors among ED nurses, examine differences in perception between nurses and patients, and explore whether selected sociodemographic factors are associated with perceived caring behaviors in the ED. **Methods**: A multicenter cross-sectional study was conducted between October 2025 and January 2026 in ten EDs in Slovenia. Descriptive and inferential statistical methods were applied. **Results**: A total of 354 nurses completed the Slovenian CBI-24, and 288 patients completed the Slovenian patient version, designated the CBI-24-P. Caring behaviors were rated highly by both nurses and patients. Among patients, the highest ratings were observed for safety-related aspects of care, such as giving medication (M = 5.42; SD = 0.85) and treating patient information confidentially (M = 5.53; SD = 0.83). Among patients, caring behaviors showed weak positive correlations with age (r_s_= 0.131) and weak negative associations with waiting time (r_s_ = −0.299). **Conclusions**: Caring behaviors in Slovenian EDs are perceived as high, with emphasis placed on technical competence. Communication, responsiveness, and workflow organization remain relevant areas for further investigation and quality improvement.

## 1. Introduction

Caring is a core concept in nursing [1,2], primarily expressed through actions and interpersonal interactions directed toward meeting patients’ needs and supporting their well-being [3]. As an integral part of nursing, caring is commonly understood as a set of observable caring behaviors enacted within the relationship between nursing staff and patients [4,5,6]. At the same time, caring is grounded in a moral and value-based framework, as it is described as both a professional commitment and a form of reciprocal human engagement [1,3,7,8].

Caring behaviors are therefore multidimensional and include both clinically oriented and relational elements [3,6,9,10]. They draw on professional knowledge, practical skills, and human attributes and are expressed through competent, accountable, and safe nursing procedures, as well as respectful and empathetic communication, emotional support, presence, responsiveness, and recognition of patients’ individual needs [1,3,7].

Conceptually, caring behaviors can be divided into two main dimensions. The first includes technical behavioral activities related to clinical skills and physical care. The second involves expressive behaviors, including emotional and psychological support, building trust, offering reassurance, fostering hope, and demonstrating compassion toward patients [3,4,11].

The present study is theoretically informed by Jean Watson’s theory of human caring, which conceptualizes caring as more than the completion of clinical tasks. Within this perspective, caring includes professional values, knowledge, moral responsibility, interpersonal engagement, and actions aimed at reducing vulnerability and preserving human dignity. Watson further defines caring as a process through which the nurse responds to another person as a unique individual, recognizes their feelings, and acknowledges their distinct human experience [12]. This framework is relevant to the study of caring behaviors because it integrates the technical, ethical, and relational dimensions of nursing and provides a basis for examining how these dimensions are maintained in complex clinical environments.

Emergency departments (EDs) represent a particularly demanding context for the delivery of caring behaviors. Care is provided under conditions characterized by urgency, time pressure, high patient turnover, overcrowding, limited resources, prolonged waiting times, delayed transfers, and nursing shortages [13,14]. Although emergency nursing is primarily oriented toward clinical stabilization, technological support, pharmacological treatment, and continuous monitoring, caring behaviors remain essential to patient safety, emotional support, comfort, clear communication, and overall well-being [15,16]. Watson’s theoretical perspective is therefore particularly relevant in this context, as it provides a framework for examining how relational and humanistic dimensions of care are sustained alongside urgent biomedical and life-sustaining interventions [12,17,18,19,20].

ED environments are designed for urgent rather than extended treatment, and patients may be unable to express their needs because of pain, altered consciousness, clinical instability, anxiety, or uncertainty [15]. In such circumstances, observation, responsiveness, and the anticipatory recognition of discomfort and other unmet needs become central expressions of caring [15,21,22].

Findings from previous studies indicate that caring behaviors in emergency nursing are most often understood in relation to clinical competence, technical performance, and patient safety [19]. The characteristics of ED care may also affect how caring behaviors are delivered and perceived. The same care encounter may be interpreted differently by nurses and patients. Nurses may evaluate caring primarily in terms of professional responsibility, clinical competence, safety, and timely intervention, whereas patients may evaluate it through the visibility of relational, communicative, and supportive behavior [20]. Patients may place greater emphasis on behaviors that are directly visible during the encounter, including clear communication, availability, responsiveness, patience, respect, emotional support, individualized attention, and whether they feel recognized as persons during waiting and treatment [1,3,23].

Caring behaviors are also shaped by individual, professional, interpersonal, and organizational factors. Nurses’ personal characteristics, attitudes, motivation, competencies, communication, and teamwork may influence the delivery of caring behaviors, while workplace resources, staffing levels, leadership, organizational culture, workload, and institutional support determine the conditions under which such behavior can be provided [3,6,9,10].

Despite contextual and cultural differences, patients across diverse settings consistently describe a ‘good’ nurse in similar terms, highlighting the importance of professional expertise alongside effective interpersonal interaction [1].

In EDs, high patient volume, time pressure, inadequate staffing, and limited managerial or institutional support can hinder the consistent provision of comprehensive, patient-centered caring behaviors [3,21,24,25]. In such conditions, nurses may need to prioritize technical and safety-related tasks, while psychosocial aspects of care, including empathy, emotional support, and patient involvement, receive less attention.

Consequently, the caring behaviors that are most visible within the clinical encounter may be those associated with technical competence, efficiency, and safety [1,3]. This is clinically relevant because caring behaviors have been associated with safer, more attentive, and higher-quality nursing care, including greater adherence to clinical protocols and safety procedures [25,26].

Previous studies have examined caring behaviors in nursing, but evidence from EDs remains limited, particularly regarding direct comparisons between nurses and patients within the same clinical setting and the extent to which these perceptions are associated with professional and organizational conditions. This limitation is important because nurses and patients may evaluate the same care encounter according to different criteria. Differences between nurses’ and patients’ assessments may reflect not only their different roles in the care encounter but also the constraints of the environment in which care is delivered.

Examining nurses’ and patients’ perspectives within the same ED may, therefore, provide a more comprehensive understanding of caring behaviors and help distinguish discrepancies related to professional roles from those arising from the organizational context. Such evidence may also support the development of interventions that address both the clinical and relational dimensions of emergency nursing care.

Accordingly, the study aimed to: (1) translate, culturally adapt, and evaluate the psychometric properties of the CBI-24 and CBI-24-P for use in the Slovenian ED context; (2) assess the level of caring behaviors among nurses and patients in EDs; (3) examine whether differences exist in the perception of caring behaviors between nurses and patients in EDs; and (4) explore whether sociodemographic factors are associated with perceived caring behaviors among patients in EDs.

## 2. Materials and Methods

### 2.1. Study Design and Setting

A multicenter cross-sectional descriptive study was conducted between October 2025 and January 2026.

This study was reported in accordance with the Strengthening the Reporting of Observational Studies in Epidemiology (STROBE) guidelines for cross-sectional studies.

### 2.2. Study Population

Eligibility criteria for both nurses and patients were defined in line with the recommendations of Amiri et al. [27] and tailored to the ED care setting.

The nurse sample comprised ED nurses meeting the following criteria: (1) completion of secondary nursing education or a higher level of nursing education; (2) active involvement in patient care in the ED; and (3) a minimum of six months of professional experience in nursing practice.

The patient sample included individuals treated in the ED who met the following criteria: (1) being 18 years of age or older; (2) proficiency in Slovenian; (3) no known cognitive or mental health disorder; (4) a duration of ED treatment of at least two hours; (5) no life-threatening condition at the time of participation; and (6) no disturbance of consciousness, severe pain, nausea, or marked fatigue during data collection.

Consecutive sampling was used. All nurses working in participating EDs across Slovenia who were available during the study period and met the predefined inclusion criteria were invited to participate until the target sample size was reached. The same recruitment procedure was applied to eligible patients treated in the participating EDs [28].

Twelve EDs operating within regional hospitals in the Republic of Slovenia were invited to collaborate in the study. Of these, ten hospitals agreed to participate in the study.

### 2.3. Data Collection

A pilot quantitative study was conducted before the main study to test the questionnaire and refine its final version. Following methodological recommendations [29], the pilot sample size was set at 20–40 participants. The questionnaire was administered to a convenience sample of 30 nurses and 30 patients at one participating ED in Slovenia, resulting in a 100% response rate. The pilot study was conducted between August and September 2025. The average completion time was 12 min for nurses and 23 min for patients.

Based on the pilot data, the minimum required sample size for the main study was calculated to ensure adequate statistical power and precision. As an estimate of the annual number of ED visits in Slovenia, data from the Ministry of Health on the number of patient visits to EDs across Slovenia in 2019 were used, amounting to 517,657 visits [30]. Based on Cochran’s formula, the minimum required patient sample size was calculated to be 221 participants using a 95% confidence level and a 5% margin of error.

Before the onset of the study, approval was obtained from the management of each participating hospital, the relevant ethics committees, and the head nurses of the EDs. The questionnaires were distributed in accordance with arrangements made with the management of each ED. In four EDs, the questionnaires were delivered in person by the researchers, and in one ED, at the request of the head nurse, the questionnaires were provided electronically (1KA). In the remaining participating EDs, the questionnaires were distributed by post according to prior arrangements. The questionnaires were administered to nurses and patients by the head nurses of the participating EDs.

Data collection was completed after the predefined study period. In one ED, completed paper questionnaires were collected in person for logistical reasons, whereas in another ED, data were obtained electronically, and access to the survey was closed after two months. In the remaining EDs, completed questionnaires were returned to the principal researcher by postal mail. Anonymity was maintained by requiring participants to return completed paper questionnaires in sealed envelopes. The principal researcher was not involved in participant identification and had no access to information linking individual questionnaires to specific respondents. All collected questionnaires were subsequently coded and assigned a unique numerical identifier. Data were manually entered into IBM SPSS Statistics (Version 28.0; IBM Corp., Armonk, NY, USA, 2021).Caring Behaviors Inventory (CBI-24)

Caring behaviors were measured using the CBI-24 in the nurse sample and its patient version, designated CBI-24-P, in the patient sample [31]. Throughout the manuscript, CBI-24 refers to the version administered to nurses, whereas CBI-24-P refers to the version administered to patients.

The CBI-24 is a standardized empirical instrument with a well-defined conceptual and theoretical framework, designed to assess perceptions of caring behaviors among patients and nurses [31]. The instrument consists of 24 items rated on a six-point Likert scale, ranging from 1 (‘never’) to 6 (‘always’) [32]. In its original structure, the CBI-24 comprises four domains: (I) Assurance of human presence (8 items) referring to actions aimed at meeting the needs and expectations of others; (II) Knowledge and skills (5 items), encompassing behavior perceived as professionally competent and skillful; (III) Respectful deference to others (6 items), including behaviors that acknowledge and validate others’ feelings; and (IV) Positive connectedness (5 items), reflecting the provision of prompt and continuous support [31].

The CBI-24 has been extensively validated internationally, demonstrating satisfactory content and external validity [32,33,34,35,36,37,38,39], and has been recognized as a reliable and valid tool for evaluating caring behaviors across diverse clinical settings [1,40,41].

The CBI-24 does not define predetermined cut-off values for categorizing levels of caring behaviors. Interpretation is therefore based on mean scores calculated within individual subscales and across the overall scale, with higher mean values indicating a higher level of perceived caring behaviors within the studied population [31,32].

For this study, the CBI-24 was translated, culturally adapted, and psychometrically analyzed in the Slovenian ED setting, resulting in three main subscales: (I) Knowledge and skills; (II) Relationally supportive and empathic caring behaviors; and (III) Assurance of human presence.

### 2.4. Translation, Cultural Adaptation, and Psychometric Evaluation of the CBI-24 and CBI-24-P

#### 2.4.1. Translation and Cultural Adaptation

The original version of the CBI-24 questionnaire had not yet been translated into Slovenian; therefore, after obtaining the authors’ permission, the back-translation method was applied. Two independent translators first translated the instrument into Slovene, and their versions were then compared and merged into a single version. A third translator subsequently translated it back into English. After completing this step, we compared the Slovene translation of the questionnaire with the original English version, resolved any discrepancies between the translations, and finalized the Slovene version of the instrument.

#### 2.4.2. Face Validity

Face validity and cultural appropriateness were examined with a convenience sample of ten nurses with substantial experience in emergency care. They reviewed each item for clarity, ease of understanding, and relevance to the intended clinical context, and suggested alternative phrasing where needed. The research team then considered all feedback and agreed on the final Slovenian wording, with the aim of ensuring both linguistic accuracy and contextual suitability for the target population.

#### 2.4.3. Content Validity

Subsequently, the content validity of the translated questionnaire was assessed using the Content Validity Index (CVI) by ten nursing experts who held a doctoral degree or were in the process of obtaining one. Each item was rated for relevance using a four-point scale where a score of 1 indicated ‘not relevant’, 2 ‘somewhat relevant’, 3 ‘quite relevant’, and 4 ‘highly relevant’ [28,42,43]. Item Content Validity Index (I-CVI), Scale Validity Index (S-CVI), Average Scale Validity Index (S-CVI/Ave), and Scale-level Content Validity Index based on Universal Agreement (S-CVI/UA) were calculated. The scores were rated good when I-CVI was ≥0.78; S-CVI/Ave was ≥0.90 [28,43,44,45], and S-CVI/UA was ≥0.8 [44,45,46]. The modified kappa statistic (κ*) was calculated to adjust for the potential influence of chance agreement. To evaluate the modified κ*, the values were classified into three categories: moderate (0.40–0.59), good (0.60–0.75), and excellent (>0.75) [47,48,49].

The I-CVI, S-CVI, S-CVI/Ave, S-CVI/UA, and κ* values were calculated using Microsoft Excel [50].

#### 2.4.4. Construct Validity

Principal Component Analysis (PCA) was performed using orthogonal varimax rotation. The Kaiser–Meyer–Olkin (KMO) index was calculated. We followed the guidelines of the authors Tabachnick and Fidell [51], whereby KMO values ≥ 0.6 indicate acceptable suitability of the data for further factor analysis, and KMO values > 0.8 indicate very good suitability. Simultaneously with the KMO, we verified the adequacy of the correlation matrix using Bartlett’s test of sphericity. In the final step of the PCA analysis, we produced a graphical representation for deciding on the number of factors of the measurement instrument (Cattell’s Scree Plot) [51,52]. We considered eigenvalues > 1.0 and included factor loadings with absolute values ≥ 0.4 [51].

We used IBM SPSS Amos (Version 26.0; IBM Corp., 2019, Armonk, NY, USA, 2019) to conduct confirmatory factor analysis, using pairwise deletion of the data. 

We used the Root Mean Square Error of Approximation (RMSEA), the chi-square to degrees-of-freedom ratio (CMIN/DF), the Incremental Fit Index (IFI), the Relative Fit Index (RFI), the Normed Fit Index (NFI), the Goodness-of-Fit Index (GFI), the Adjusted Goodness-of-Fit Index (AGFI), and the Comparative Fit Index (CFI). Acceptable model fit was defined as RMSEA < 0.10, CMIN/DF < 3, and IFI, RFI, NFI, GFI, AGFI, and CFI values ≥ 0.90 [52].

#### 2.4.5. Internal Consistency

Internal consistency was assessed with Cronbach’s α and McDonald’s ω. The Cronbach’s α coefficient was judged based on the recommendations of Tavakol and Dennick [53]: unacceptable (α < 0.50), poor (0.50 ≤ α < 0.60), questionable (0.60 ≤ α < 0.70), acceptable (0.70 ≤ α < 0.90), and excellent (α ≥ 0.90).

For the interpretation of McDonald’s ω values, we followed the recommendations of Hayes and Coutts [54]: insufficient reliability (ω < 0.60); marginal reliability (0.60 < ω < 0.69); acceptable reliability (0.70 < ω < 0.79); good reliability (0.80 < ω < 0.89); and excellent reliability (ω ≥ 0.90).

### 2.5. Statistical Analysis

Descriptive and inferential statistical methods were applied. Descriptive statistics included frequency, minimum (min) and maximum (max) values, standard deviation (SD), mean (M), and 95% confidence intervals. Statistical significance was set at *p* < 0.05.

Both parametric and non-parametric tests were used in the inferential analysis, with the choice of test based on prior assessment of data distribution. The arithmetic mean difference between nurses’ and patients’ ratings was calculated as ΔM = M (nurses) − M (patients), with positive values indicating higher mean scores among nurses and negative values indicating higher mean scores among patients.

Normality was assessed using visual inspection of histograms and the Kolmogorov–Smirnov or Shapiro–Wilk tests, with selection depending on sample size. Differences in perceived caring behaviors according to demographic characteristics were examined using the Mann–Whitney U test or Kruskal–Wallis test. Distributions were considered non-normal when the normality tests were statistically significant, with skewness, kurtosis, and visual inspection also taken into account [55].

To evaluate the magnitude of group differences in each domain and in the total score, effect-size measures appropriate for the non-parametric statistical tests were calculated. For demographic and clinical variables with two independent groups, the Mann–Whitney U-test was used, and the effect size was expressed as Rosenthal’s r, calculated as $r=|Z|/\sqrt{N}$, where Z represents the standardized test statistic and N the total number of observations included in the comparison. Values were interpreted as follows: small (0.10 ≤ |r| < 0.30), medium (0.30 ≤ |r| < 0.50), and large (|r| ≥ 0.50) [56].

For variables with more than two independent groups, the Kruskal–Wallis test was used, and ordinal eta-squared (η^2^H) was calculated as η^2^H = (H − k + 1)/(N − k), where H represents the Kruskal–Wallis test statistic, k is the number of groups, and N is the total number of observations across all groups. Values were interpreted small (0.01 ≤ η^2^H < 0.06), medium (0.06 ≤ η^2^H < 0.14), and large (η^2^H ≥ 0.14) [56,57].

Spearman’s rank correlation coefficient (r_s_) was used to examine associations between caring behaviors and selected variables, including age, years of experience, time to initial physician assessment, and treatment duration. In interpreting the correlation coefficients, we followed the criteria proposed by Dancey and Reidy [58] based on the absolute value of r_s_: |r_s_| = 1.000 a perfect correlation; 0.700 < |r_s_| < 1.000 a very strong correlation; 0.400 < |r_s_| ≤ 0.700 a moderate correlation; 0.100 < |r_s_| ≤ 0.400 a weak correlation; and |r_s_| < 0.100 indicated no correlation [59].

Statistical analyses were conducted using IBM SPSS Statistics version 28.0, and the results were subsequently processed and reported in Microsoft Word 2019.

## 3. Results

### 3.1. Translation, Cultural Adaptation, and Psychometric Evaluation of the CBI-24 and CBI-24-P

#### 3.1.1. Translation and Cultural Adaptation

The translation and back-translation process resulted in Slovenian versions of the CBI-24 and CBI-24-P that were semantically consistent with the original English instrument. Comparison of the back-translated and original versions revealed no substantial discrepancies in item meaning. Minor linguistic differences were resolved through discussion among the translators and the research team, resulting in final Slovenian versions that preserved the intended meaning of the original items while ensuring linguistic and cultural appropriateness for the Slovenian emergency care context.

#### 3.1.2. Face Validity

Based on feedback from the participating nurses, minor clarifications and cultural adaptations were recommended, while all items remained in the CBI-24 instrument.

#### 3.1.3. Content Validity

A panel of ten PhD-qualified nursing experts evaluated the Slovenian CBI-24 for item relevance and overall suitability in emergency care. Their ratings were used to calculate item- and scale-level content validity indices. As no item was considered problematic, the questionnaire was retained without further changes or item removal.

Content validity indices (CVI) indicated high agreement. The S-CVI/Ave values ranged from 0.983 for the CBI-24-P questionnaire to 1.000 for the CBI-24 questionnaire. Similarly, the S-CVI/UA values ranged from 83.33% for the patient questionnaire (CBI-24-P) to 100% for the nurse questionnaire (CBI-24).

For the CBI-24 administered to nurses, the I-CVI value for every item was 1.00, indicating excellent content validity. The overall CVI of the inventory was calculated to be 1, which is at an excellent level. Furthermore, the modified kappa coefficients of all 24 items were reported to be 1, which is excellent (Table A5).

For the CBI-24-P administered to patients, the CVIs of all items ranged from 0.90 to 1.00 and were therefore considered acceptable. The overall CVI of the inventory was calculated to be 0.983, indicating excellent content validity. Furthermore, the modified kappa coefficients ranged from 0.899 to 1.00 for all 24 items, indicating excellent agreement among the experts (Table A6).

#### 3.1.4. Construct Validity

As the CBI-24 had not yet been adapted to the Slovenian emergency nursing context, the instrument was tested and validated prior to its use in the study.

The Kaiser–Meyer–Olkin (KMO) index for CBI-24 among nurses was excellent at 0.955, indicating very good sampling adequacy. Bartlett’s test of sphericity was statistically significant for the CBI-24 (χ^2^ = 6731.967, df = 276, *p* < 0.001). The Kaiser–Meyer–Olkin (KMO) index for CBI-24-P among patients was excellent at 0.958, indicating very good sampling adequacy. Bartlett’s test of sphericity was also statistically significant for the CBI-24-P (χ^2^ = 6063.757, df = 276, *p* < 0.001).

In the nurse sample, PCA identified a three-component solution for the CBI-24, explaining 66.63% of the total variance. The first subscale, named Knowledge and skills (items 10, 9, 11, 21, 23, 15, 12, 24, 3, 2, and 22), explained 54.27% of the total variance (Table A3). The second subscale was named Relationally supportive and empathic caring behaviors (items 5, 7, 4, 6, 8, and 1) and explained 7.81% of the variance. The third subscale was labeled Assurance of human presence (items 19, 16, 18, 17, 20, 14, and 13), explaining 4.55% of the total variance (Table A4).

In the patient sample, PCA identified a three-component solution for the CBI-24-P, accounting for 69.99% of the variance (Table A1). In the CBI-24-P, the first subscale (Assurance of human presence) explained 58.96% of the total variance and included items 17, 23, 24, 20, 22, 18, 19, 21, and 16. The second subscale (Relationally supportive and empathic caring behaviors) explained 5.92% and comprised items 2, 1, 7, 6, 3, 5, 4, 14, 13, and 8. The third subscale (Knowledge and skills) included items 12, 10, 9, 15, and 11 and explained 5.11% of the variance (Table A2).

We compared four models:

(I). The original four-factor CBI-24 model in the nurse sample with 24 items and 4 subscales: Assurance of human presence (8 items), Knowledge and skills (5 items), Respectful deference to others (6 items), and (IV) Positive connectedness (5 items).
(II). The Slovenian three-factor CBI-24 model in the nurse sample with 24 items and 3 subscales: Knowledge and skills (11 items), Relationally supportive and empathic caring behaviors (6 items), and Assurance of human presence (7 items).
(III). The original four-factor CBI-24 model in the patient sample with 24 items and 4 subscales: Assurance of human presence (8 items), Knowledge and skills (5 items), Respectful deference to others (6 items), and (IV) Positive connectedness (5 items).
(IV). The Slovenian three-factor CBI-24-P model in the patient sample with 24 items and 3 subscales: Knowledge and skills (5 items), Relationally supportive and empathic caring behaviors (10 items), and Assurance of human presence (9 items). Detailed PCA results are presented in Appendix A (Table A1, Table A2, Table A3 and Table A4).

In the nurse sample, the original four-factor CBI-24 model (Assurance of human presence, Knowledge and skills, Respectful Deference to Others, and Positive Connectedness) demonstrated poor fit to the data. The RMSEA was 0.158 and the CMIN/DF ratio was 9.813, both substantially exceeding the predefined acceptable values. All remaining fit indices were also below 0.90: IFI = 0.666, RFI = 0.608, NFI = 0.642, GFI = 0.640, AGFI = 0.610, and CFI = 0.665. These findings indicate that the original four-factor structure did not adequately represent the covariance structure of the Slovenian nurse data.

In contrast, the three-factor Slovenian CBI-24 model demonstrated acceptable overall fit in the nurse sample: χ^2^(249) = 704.66, *p* < 0.001, CMIN/DF = 2.83, RMSEA = 0.072, IFI = 0.914, and CFI = 0.912. The RFI = 0.872, NFI = 0.891, GFI = 0.889, and AGFI = 0.864 were slightly below the indicative value of 0.90. Therefore, although not all fit indices reached the recommended threshold, the combination of an acceptable RMSEA and CMIN/DF ratio and IFI and CFI values above 0.90 supported an acceptable and substantially improved fit compared with the original four-factor model.

A comparable pattern was observed in the patient sample. The original four-factor CBI-24 model (Assurance of human presence, Knowledge and skills, Respectful Deference to Others, Positive Connectedness) demonstrated poor fit, with RMSEA = 0.162 and CMIN/DF = 8.531. The IFI = 0.705, RFI = 0.617, NFI = 0.678, GFI = 0.690, AGFI = 0.630, and CFI = 0.702 were all below the predefined acceptable value.

By comparison, the three-factor Slovenian patient version, CBI-24-P, demonstrated good overall fit to the data: χ^2^(75) = 106.08, *p* = 0.011, CMIN/DF = 1.41, RMSEA = 0.038, IFI = 0.992, RFI = 0.906, NFI = 0.970, GFI = 0.940, AGFI = 0.910, and CFI = 0.970. All reported fit indices met the predefined criteria for acceptable model fit.

Taken together, the CFA results did not support the original four-factor structure in either sample. The three-factor solutions provided substantially better fit, with acceptable fit in the nurse sample and good fit in the patient sample. These findings support the use of the Slovenian three-factor CBI-24 and CBI-24-P structures in the present study. A list of the most important CFA goodness-of-fit indices is provided in Appendix A (Table A7).

#### 3.1.5. Internal Consistency

In the ED nurse sample, the CBI-24 demonstrated excellent internal consistency. Cronbach’s α was 0.95 for Knowledge and skills, 0.89 for Relationally supportive and empathic caring behaviors, and 0.90 for Assurance of human presence. Cronbach’s α and McDonald’s ω for the overall CBI-24 were both 0.96.

In the ED patient sample, the CBI-24-P demonstrated excellent internal consistency, with Cronbach’s α and McDonald’s ω values of 0.97 for the overall questionnaire. Cronbach’s α values in CBI-24-P reached 0.85 for Knowledge and skills, 0.95 for Relationally supportive and empathic caring behaviors, and 0.95 for Assurance of human presence.

Overall, these findings demonstrate high internal consistency of the CBI-24 and CBI-24-P and their respective subscales.

### 3.2. Sociodemographic Characteristics

#### 3.2.1. Nurses

The study sample comprised 354 nurses in EDs, with a predominance of women (78.5%) compared to men (21.5%). The mean age of the participants was 36.80 years (SD = 10.19). Regarding educational attainment, most participants held a bachelor’s degree (66.7%), followed by secondary school education (22.3%) and a master’s degree (10.7%).

The mean total years of experience was 14.59 (SD = 10.76). In EDs, most participants had up to 5 years of experience (47.7%), followed by 6–10 years (26.0%), with fewer reporting longer tenure. The mean duration of experience in EDs was 9.24 years (SD = 9.17) (Table 1). Among ED nursing staff, 519 questionnaires were distributed, of which 354 were returned and appropriately completed, corresponding to a response rate of 68.2%. Among patients, 445 questionnaires were distributed, of which 288 were returned and appropriately completed, corresponding to a response rate of 64.7%.

#### 3.2.2. Patients

The patient sample comprised 288 individuals, with a slight predominance of women (52.1%) compared to men (47.9%). The sample included patients across a wide age range, with the highest proportions in those over 70 years (20.5%) and 41–50 years (19.8%), followed by 18–30 years (19.1%) and 51–60 years (17.0%), while the 31–40 (12.5%) and 61–70 (11.1%) groups were less represented.

The mean age of the participants was 50.66 (SD = 19.40). Regarding admission to the ED, most patients were accompanied by family members (45.1%), followed by those arriving independently (29.5%) or by ambulance (22.9%), while only a small proportion arrived from another ward (2.4%). Most patients reported total treatment times longer than 4 h (34.7%), followed by 3–4 h (23.3%) and 2–3 h (21.5%), while shorter durations were less common, including 1–2 h (15.0%) and less than one hour (5.6%). Most patients expressed a positive inclination to recommend the visited ED to family and friends, with 66.3% responding ‘absolutely’ and 27.8% ‘maybe,’ while only a small proportion were uncertain (4.5%) or would not recommend it (1.4%) (Table 2).

### 3.3. Comparative Evaluation of Caring Behaviors in the ED: Patient and Nurse Perspectives

Table 3 summarizes patients’ and nurses’ reported evaluations of caring behaviors among ED nurses, demonstrating consistently high ratings, with most mean values clustering at or above 5 on a six-point scale.

The highest scores among patients were observed in domains reflecting technical competence, safety, and reliability of care, including skillful use of medical equipment (M = 5.49; SD = 0.76), confident interaction with patients (M = 5.29; SD = 0.85), and timely delivery of treatments and medications (M = 5.29; SD = 1.02). Interpersonal aspects of care were also rated favorably, particularly attentive listening (M = 5.27; SD = 1.00), clarity of communication and patient education (M = 5.21; SD = 0.94), and empathic engagement (M = 5.05; SD = 1.21). In contrast, comparatively lower ratings were assigned to items related to time availability and patient involvement, including time spent addressing patient needs (M = 4.84; SD = 1.28), inclusion in care planning (M = 4.83; SD = 1.35), and nurses’ unsolicited return to the patient (M = 4.81; SD = 1.44).

Among nurses, the highest-rated items were related to confidentiality and professional competence, particularly treating patient information confidentially (M = 5.69; SD = 0.72), administering medications and procedures such as injections and infusions (M = 5.59; SD = 0.85), reducing patient pain (M = 5.54; SD = 0.81), and providing treatments promptly (M = 5.47; SD = 0.85). High scores were also observed in interpersonal domains, including attentive listening (M = 5.37; SD = 0.81), empathic engagement (M = 5.32; SD = 0.88), and communication with the patient (M = 5.35; SD = 0.92). In contrast, lower mean values were noted for items related to time availability and developmental support, particularly spending sufficient time with the patient (M = 4.84; SD = 1.10) and supporting patient growth (M = 4.53; SD = 1.24).

As shown in Table 3, nurses generally reported higher mean scores than patients across most items, although the differences were small. The largest gaps were observed in patience, responsiveness, communication, and time-related aspects of care, where patients consistently provided lower ratings (Table 3).

### 3.4. Differences in Perceived Caring Behaviors According to Demographic Characteristics

Analysis of mean domain scores showed that the highest values in Knowledge and skills were reported by women (5.53 ± 0.62), nurses with a bachelor’s degree (5.53 ± 0.57), those aged 51–60 years (5.66 ± 0.38), with 11–15 years of total work experience (5.58 ± 0.48), and with more than 21 years of ED experience (5.61 ± 0.55). A similar pattern was observed for Relationally supportive and empathic caring behaviors and Assurance of human presence, with the highest ratings among women, older nurses, and those with longer work experience.

Based on the total CBI-24 score, the highest mean values were observed among women (5.34 ± 0.65), nurses with secondary education (5.33 ± 0.58), those aged 51–60 years (5.46 ± 0.49), with 11–15 years of experience (5.40 ± 0.47), and those with more than 21 years of ED experience (5.43 ± 0.56) (Table 4).

Statistically significant differences in nurses’ evaluations of caring behaviors in the ED were observed primarily by sex and age, as shown in Table 5. Female nurses reported higher ratings across all CBI-24 domains: Knowledge and skills (U = 8946.00; *p* = 0.039; r = 0.11), Relationally supportive and empathic caring behaviors (U = 7817.00; *p* < 0.001, r = 0.19), and Assurance of human presence (U = 7685.50; *p* < 0.001, r = 0.20). A significant age-related difference was observed in Relationally supportive and empathic caring behaviors, with respondents aged 41–50 years reporting higher scores (χ^2^ = 11.560; *p* = 0.021; η^2^H = 0.022). For the overall caring-behaviors score, a statistically significant difference was found only by sex (U = 7849.00; *p* < 0.001; r = 0.18), with higher ratings among women. However, all effect sizes were small, indicating that the magnitude of these differences was limited. No statistically significant differences were observed according to educational level, total years of work experience, or years of experience in ED.

Based on the overall CBI-24-P score, the highest mean ratings were reported by male patients (5.27 ± 0.77), those referred from another department (5.83 ± 0.26), those who waited less than 30 min (5.39 ± 0.82), and those with total care duration up to two hours (5.46 ± 0.59). The lowest mean score was observed among patients waiting more than 120 min (4.50 ± 1.09). Higher ratings were also reported by patients aged 61–70 years (M = 5.38; SD = 0.74) and those aged over 70 years (M = 5.23; SD = 0.81) (Table 6).

Statistically significant differences in patients’ evaluations of caring behaviors in the ED were observed primarily for mode of arrival, waiting time for initial physician assessment, and total duration of care, as shown in Table 7. The Kruskal–Wallis test indicated significant differences by mode of arrival in the domains of Relationally supportive and empathic caring behaviors (χ^2^ = 10.803; *p* = 0.013; η^2^H = 0.031), professional Knowledge and skills (χ^2^ = 8.749; *p* = 0.033; η^2^H = 0.022), and in the overall caring-behaviors score (χ^2^ = 7.898; *p* = 0.048; η^2^H = 0.023). All corresponding effect sizes were small.

Time to initial physician assessment was significantly associated with all three caring-behaviors domains and the overall score: Knowledge and skills (χ^2^ = 27.672, *p* < 0.01, η^2^H = 0.084), Relationally supportive and empathic caring behaviors (χ^2^ = 34.143, *p* < 0.01, η^2^H = 0.107), Assurance of human presence (χ^2^ = 24.734, *p* < 0.01, η^2^H = 0.073), and the overall caring-behaviors score (χ^2^ = 32.506, *p* < 0.01, η^2^H = 0.101). The effect sizes for these differences were medium.

Overall treatment duration was significantly associated with Knowledge and skills (χ^2^ = 16.242, *p* < 0.01, η^2^H = 0.043), Relationally supportive and empathic caring behaviors (χ^2^ = 14.327, *p* < 0.01, η^2^H = 0.036), and the overall caring-behaviors score (χ^2^ = 13.150, *p* < 0.05, η^2^H = 0.032). These effect sizes were small. No statistically significant difference was found for Assurance of human presence. No statistically significant differences in caring-behaviors scores were observed according to patients’ sex or age (Table 7).

### 3.5. Differences in the Perception of Caring Behaviors Between ED Nurses and Patients

ED nurses using the CBI-24 rated caring behaviors highly across the corresponding 24 items, with mean scores predominantly exceeding 5 on the rating scale. Despite this overall positive evaluation, a consistent pattern emerged: nurses generally had higher mean ratings than patients across most items.

**Figure 1:**
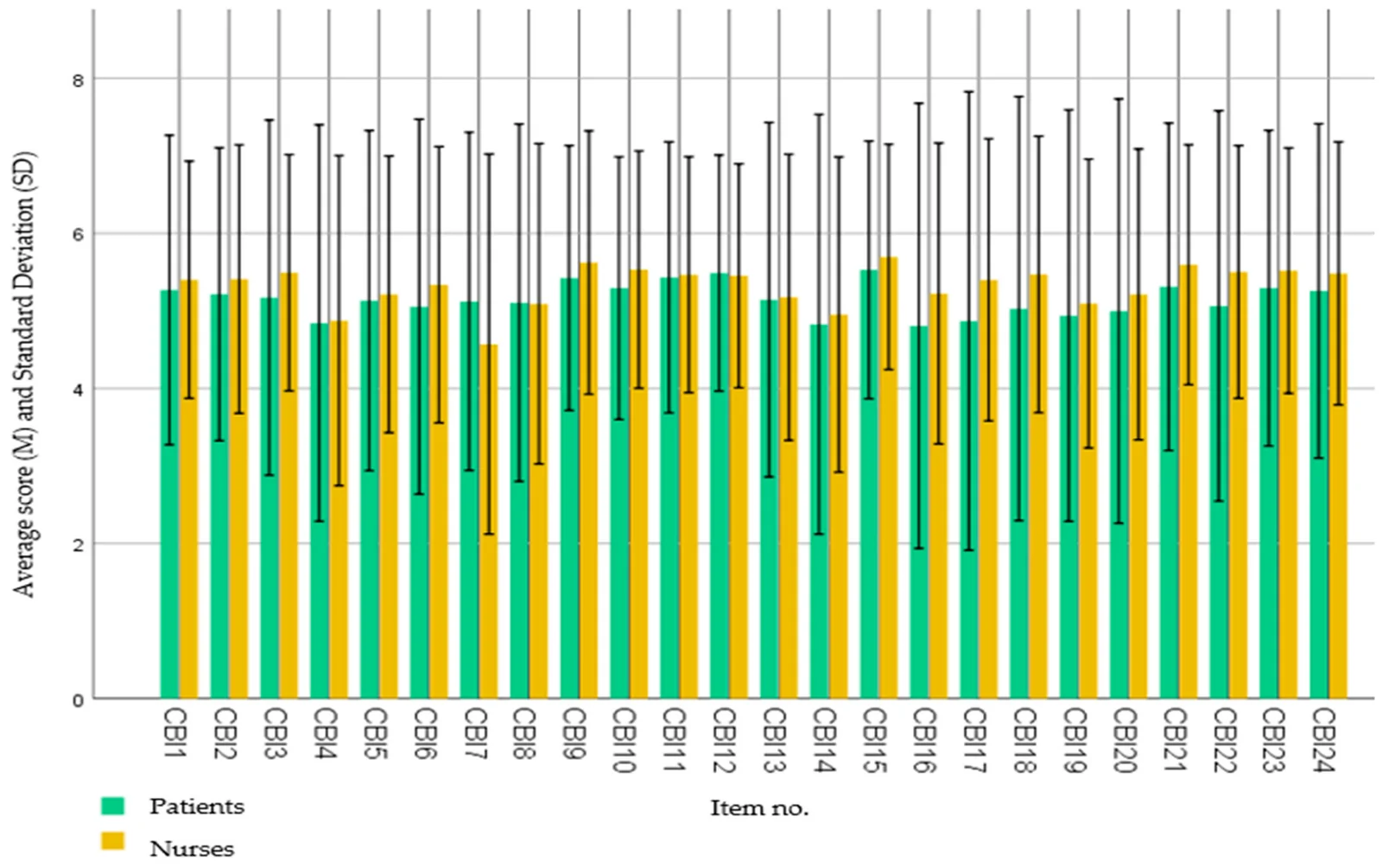
Comparison of caring behaviors ratings between ED patients and nurses.

Differences were minimal for items related to provision of support (CBI5: ΔM = 0.05), patient involvement in care planning (CBI14: ΔM = 0.08), professional Knowledge and skills (CBI11: ΔM = 0.01), and skillful use of equipment (CBI12: ΔM = 0.09), where mean scores between the two groups were nearly identical. Slightly larger differences were observed for empathic engagement (CBI6: ΔM = 0.27), treating patients as individuals (CBI3: ΔM = 0.29), returning to the patient voluntarily (CBI16: ΔM = 0.37), communication with the patient (CBI17: ΔM = 0.48), encouraging the patient to call if problems occurred (CBI18: ΔM = 0.39), and showing concern for the patient (CBI22: ΔM = 0.39). In most of these domains, patients reported lower ratings than nurses. Standard deviations were generally higher among patients (Figure 1).

Among nurses, age showed weak positive correlations with Knowledge and skills (r_s_ = 0.129; *p* < 0.05), Relationally supportive and empathic caring behaviors (r_s_ = 0.140; *p* < 0.01), Assurance of human presence (r_s_ = 0.151; *p* < 0.05), and the total CBI-24 score (r_s_ = 0.147; *p* < 0.05). Total work experience was weakly positively correlated with Knowledge and skills (r_s_ = 0.108; *p* < 0.05), Relationally supportive and empathic caring behaviors (r_s_ = 0.104; *p* < 0.05), and the total CBI-24 score (r_s_ = 0.107; *p* < 0.05). Years of experience in the ED showed a weak positive correlation only with Knowledge and skills (r_s_ = 0.107; *p* < 0.05) and was not significantly associated with the other domains or the total score.

In the patient sample, age showed weak positive correlations with Relationally supportive and empathic caring behaviors (r_s_ = 0.111; *p* < 0.01), Assurance of human presence (r_s_ = 0.161; *p* < 0.01), and the total CBI-24-P score (r_s_ = 0.131; *p* < 0.01). In contrast, longer time to initial physician assessment and longer treatment duration were weakly associated with lower ratings of caring behaviors across all domains and the total score (r_s_= −0.163 to −0.299, all *p* < 0.01). Although these associations were statistically significant, their magnitudes were small and should be interpreted cautiously (Table 8).

A multiple linear regression analysis was conducted to examine whether patients’ gender, age, time to initial physician assessment, overall treatment duration, and ED mode of arrival were associated with the total CBI-24-P score. The multiple linear regression model was statistically significant, F(5282) = 8.55, *p* < 0.001, explaining approximately 13.2% of the variance in patients’ total caring-behaviors scores (R = 0.363, R^2^ = 0.132; adjusted R^2^ = 0.116). The standard error of the estimate was 0.828.

Time to initial physician assessment was the strongest standardized predictor in the model and was negatively associated with the total CBI-24-P score (B = −0.145, SE = 0.038, β = −0.226, t = −3.845, *p* < 0.001, 95% CI for B [−0.219, −0.071]). Longer waiting times were therefore associated with lower patient ratings of caring behaviors.

Overall treatment duration was also negatively associated with the total CBI-24-P score (B = −0.105, SE = 0.042, β = −0.149, t = −2.505, *p* = 0.013, 95% CI for B [−0.188, −0.023]). Longer treatment duration was associated with lower total caring-behaviors scores.

Age was positively associated with the total score (B = 0.065, β = 0.131, 95% CI for B [0.009, 0.122], *p* = 0.023), whereas gender showed a negative association (B = −0.215, β = −0.122, 95% CI for B [−0.409, −0.021], *p* = 0.030). Thus, higher age values were associated with higher perceived caring-behaviors scores, while controlling for the remaining variables. The ED mode of arrival was not statistically significantly associated with the total CBI-24-P score (B = 0.092, β = 0.081, 95% CI for B [−0.035, 0.218], *p* = 0.155 (Table 9).

The Durbin–Watson statistic was 1.612, which does not suggest substantial first-order autocorrelation of the residuals.

A multiple linear regression analysis was conducted to examine whether nurses’ age, gender, total years of professional experience, and years of experience in the ED were associated with the total CBI-24 score. The multiple linear regression model was statistically significant, F((3350) = 36.72, *p* < 0.001, explaining approximately 23.9% of the variance in nurses’ total caring-behaviors scores (R = 0.489; R^2^ = 0.239; Adjusted R^2^ = 0.233). The standard error of the estimate was 0.867.

Years of experience in the ED specifically was also significantly associated with the total CBI-24 score, but in the opposite direction (B = −0.498, SE = 0.108, β = −0.312, t = −3.506, *p* < 0.001, 95% CI for B [−0.487, −0.137]). More years of ED-specific experience were therefore associated with lower self-rated caring-behaviors scores.

Gender was also significantly associated with the total score (B = 6.845, SE = 1.912, β = 0.178, t = 3.580, *p* < 0.001, 95% CI for B [3.086, 10.604]). Age was not statistically significantly associated with the total CBI-24 score (B = 0.198, β = 0.127, 95% CI for B [−0.095, 0.491], *p* = 0.185) (Table 10). The Durbin–Watson statistic was 1.943, which does not suggest substantial first-order autocorrelation of the residuals (Table 10).

## 4. Discussion

This study examined caring behaviors in Slovenian EDs from the perspectives of both nurses and patients. Specifically, it assessed the perceived level of caring behaviors, compared evaluations between the two groups, and explored associations with selected sociodemographic factors. In addition, the Slovenian versions of the CBI-24 for nurses and patients were cross-culturally adapted and psychometrically evaluated to ensure their appropriateness for use in the Slovenian emergency care context.

The translation and cultural adaptation process resulted in Slovenian versions of the CBI-24 and CBI-24-P that demonstrated satisfactory psychometric properties in the ED context.

The original four-factor structure did not adequately fit the data in either sample, whereas the three-factor solutions demonstrated substantially better fit. For nurses, the three-factor model showed acceptable overall fit, although several indices remained slightly below the conventional threshold of 0.90, indicating that the model was improved but not uniformly strong across all fit criteria. For patients, the three-factor CBI-24-P model met all predefined criteria and demonstrated good overall fit. Taken together, these findings support the three-factor structures identified in the Slovenian samples rather than the original four-factor model. The different factor solutions may reflect the context-specific and perspective-dependent nature of caring behaviors in EDs, where nurses may interpret caring primarily through professional competence and clinical responsibility, whereas patients may place greater emphasis on presence, responsiveness, and relational support. Importantly, the findings indicate that caring behaviors in emergency nursing may be structured differently across cultural and clinical contexts, underscoring the need for context-specific psychometric validation rather than uncritical reliance on the original model. To place these findings in a broader context, previous validation studies of the CBI-24 have reported factor structures ranging from one to four domains across different countries and clinical settings. A three-factor structure was also identified in China (Cronbach’s α 0.98 for patients and 0.97 for nurses), comprising respect and connectedness, Knowledge and skills, and support and reassurance [59], and Korea (Cronbach’s α = 0.95), where the subscales included empathic relationship and emotional support (Cronbach’s α = 0.93), Knowledge and skills (Cronbach’s α = 0.86), and comfort provision (Cronbach’s α = 0.85) [60]. Furthermore, a study conducted in the United States [39] reported a two-factor solution (Cronbach’s α = 0.97) explaining 64.02% of the variance, with highly similar factor loadings suggesting a potential one-factor structure, further supporting the variability of factor solutions across different contexts. Wu et al. [31] reported four subscales: Assurance of presence (Cronbach’s α = 0.92), Knowledge and skills (α = 0.87 for patients; α = 0.83 for nurses), Respectfulness (α = 0.91 for patients; α = 0.92 for nurses), and Positive connectedness (α = 0.82 for patients; α = 0.87 for nurses). Comparable internal consistency values were reported by Khaletabad et al. [35] with Cronbach’s α of 0.95. In that study, item 19 was excluded, as it would have reduced internal consistency to Cronbach’s α = 0.32. High internal consistency of the CBI-24 has also been reported across multiple countries, such as Iran (0.93) [2], Turkey (0.99 for patients and 0.97 for nurses) [34], Ethiopia (0.89) [5] and Indonesia (0.812) [27].

As mentioned, the present findings suggest that the structure of caring behaviors differs according to respondent perspective. Although a three-component structure was identified in both samples, nurses and patients organized the CBI-24 items differently. In the nurse sample, the strongest component was Knowledge and skills, including 11/24 items (54.27% of the variance), followed by Relationally supportive and empathic caring behaviors, including 6/24 items (7.81% of the variance), and Assurance of human presence, including 7/24 items (4.55% of the variance). This indicates that nurses primarily interpreted caring behaviors through professional competence, technical performance, patient safety, and clinical responsibility. In contrast, in the patient sample, the strongest component was Assurance of human presence, including 9/24 items (58.96% of the variance), followed by Relationally supportive and empathic caring behaviors, including 10/24 items (5.92% of the variance), and Knowledge and skills, including 5/24 items (5.11% of the variance). This suggests that patients mainly experienced caring behaviors through nurses’ presence, responsiveness, reassurance, availability, and active attention to their needs.

The findings of the present study indicate that nurses rated their caring behaviors slightly higher than patients. The most pronounced differences were identified in relational and responsiveness-related behaviors. Nurses nevertheless rated most caring behaviors more favorably than patients. The largest mean differences were observed for talking to the patient (ΔM = 0.48), encouraging the patient to call when problems occurred (ΔM = 0.39), showing concern for the patient (ΔM = 0.39), and returning to the patient voluntarily (ΔM = 0.37). Nurses also rated treating patients as individuals and demonstrating empathy more favorably than patients. By contrast, patients provided slightly higher ratings for managing equipment skillfully and demonstrating professional Knowledge and skills, suggesting that technical competence was more directly recognized and consistently experienced than some interpersonal aspects of care. Although some differences were statistically significant, the corresponding effect sizes were small, indicating limited practical importance. The findings suggest that nurses and patients evaluate caring from partly different perspectives rather than demonstrating substantial differences in the overall level of care. Results from our study are consistent with similar studies reporting higher self-assessment among nurses [2,36]. However, these results differ from studies [27,61,62,63] where patients reported more favorable evaluations of caring behaviors than nurses.

Alikari et al. [1] similarly identified professional Knowledge and skills as highly rated dimensions of caring while emphasizing empathy and supportive communication as essential interpersonal components. In the present study, greater variability in patient responses and lower ratings of communication and responsiveness suggest that relational aspects of care may be experienced less consistently than technical care [2].

These statistically significant findings indicate that patients who experienced longer overall treatment durations tended to report lower levels of relationally supportive and empathic caring behaviors, perceived knowledge and skills, and overall caring behaviors. Although several differences were statistically significant, the corresponding effect sizes were small. Their practical importance therefore appears limited, and clinical relevance cannot be inferred from statistical significance alone. The observed discrepancies should consequently be interpreted as modest differences in perception rather than as evidence of substantial differences in the quality of care provided.

In the study by Enns et al. [21], caring was related to several factors, including workload, lack of time, staffing issues, shift work, and lack of self-care. Azizi-Fini et al. [64] emphasized that caring behaviors are not limited to clinical tasks but also include attitudes and approaches toward patients, with particular importance given to respect for patient individuality. Differences between nurses’ and patients’ perceptions may reflect distinct evaluative perspectives. Nurses are likely to assess caring behaviors in relation to professional standards, clinical competence, patient safety, and task completion, whereas patients evaluate care through their immediate experience of communication, responsiveness, reassurance, and staff availability [27,64]. This interpretation is supported by the present findings, as the most pronounced discrepancies concerned relational and availability-related behaviors, while evaluations of technical competence were largely similar. Another challenge in the ED is the high number of patients, including many with non-urgent conditions, which increases workload and reduces time for individual care. Because of this, nurses do not always have enough time to be with patients, stay available, or offer continuous support [65,66].

Patient evaluations were associated with selected organizational and clinical characteristics of care. In the multivariable regression model, longer time to initial physician assessment and longer overall treatment duration were independently associated with lower total CBI-24-P scores after adjustment for sex, age, and mode of arrival. Time to initial physician assessment showed the strongest standardized association, whereas the association with treatment duration was weaker. These findings indicate that waiting time and treatment duration were related to patients’ perceptions of caring.

Caring is a multidimensional concept that integrates clinical, emotional, and ethical components of nursing practice [1,19]. Professional nursing practice requires the consistent application of caring behaviors in patient care. When these are lacking—such as through insufficient attention, inappropriate treatment, or delayed and non-standardized care—negative consequences may arise for patients, nurses, and the healthcare institution. In such cases, patients may perceive the quality of care more critically, which can reduce trust in the institution and influence their willingness to seek care there in the future [67]. The findings highlight the need for comprehensive strategies to strengthen caring behaviors in emergency nursing. These differences have practical implications for emergency nursing. Interventions should emphasize concise, patient-centered communication, regular updates during waiting, clear explanations of delays and procedures, timely responses to patients’ needs, and intentional follow-up whenever clinical circumstances allow. At the organizational level, improvements in workflow, staffing allocation, and waiting-time management may help support more consistent relational and patient-centered care in demanding emergency settings.

### 4.1. Limitations

The study has several limitations. The use of a convenience sample and cross-sectional design limits the generalizability of the findings. In addition, the sample included only nurses working in EDs; therefore, the findings cannot be generalized to the broader population of healthcare professionals in other clinical departments. A further limitation was the lack of participation from some invited hospitals or EDs, because they declined or did not respond to the invitation.

Another limitation of this study was the smaller patient cohort compared with the nurses’ cohort, which may be explained by challenges associated with data collection in the emergency care setting. Even though questionnaires were distributed to a larger number of patients presenting to the ED, not all were returned, as some patients forgot to return them after discharge and some questionnaires were incomplete and therefore excluded from the final analysis. In EDs, recruitment can be particularly challenging due to patients’ acute health conditions, stress, and time constraints, all of which may influence participation.

An important limitation of this study is related to the patient sampling strategy. Patients with life-threatening conditions, altered consciousness, severe pain, nausea, or marked fatigue were excluded because they were not clinically or ethically suitable for completing a self-report questionnaire during emergency treatment. Consequently, the patient sample mainly reflects clinically stable, cooperative, and communicatively able ED patients rather than the full spectrum of ED users. More acutely ill or vulnerable patients may experience caring behaviors differently, particularly in relation to nurses’ presence, responsiveness, communication, emotional support, and pain-related care. Self-report measurement may also be affected by social desirability among nurses, recall or situational distress among patients, and ceiling effects arising from generally high scores. The use of a single instrument means that some relevant dimensions of caring and patient experience may not have been captured. Therefore, the findings should be generalized with caution and should not be interpreted as being representative of patients with the most severe or unstable clinical conditions in EDs.

Because participants were nested within 10 EDs, responses from individuals within the same site may have been correlated; as clustering was not assessed using intraclass correlation coefficients or accounted for through multilevel modelling, the independence assumption may have been violated, potentially resulting in underestimated standard errors and overstated statistical significance of some individual-level associations.

As only a single instrument was used to assess caring behaviors, it is possible that not all dimensions of the research problem were fully captured. A further limitation of this study may be that, due to its cross-sectional design, it does not capture long-term effects or allow for monitoring changes in the perception of caring behaviors over time. In addition, the study was conducted during the winter period, when patient inflow, particularly due to viral illnesses, was substantially higher, which may have influenced the results, as nurses were exposed to increased workload and ED visits were considerably elevated.

### 4.2. Strengths

The study showed differences in specific domains of caring behaviors between nurses and patients, providing insight into the perceptions and needs of both groups. A key strength is that this is the first study conducted in Slovenian EDs using the CBI-24 questionnaire among both nurses and patients. The research was carried out in ten of the twelve EDs, covering most tertiary healthcare institutions in Slovenia. In addition, the study included a large sample, which supports the reliability of the findings. The use of a validated and culturally adapted instrument ensured consistent measurement of caring behaviors. An important advantage is the direct comparison of nurses’ and patients’ perspectives, offering a more complete view of caring in practice. The inclusion of factors such as waiting time and duration of treatment furthers our understanding of how care is experienced in real emergency settings.

## 5. Conclusions

The Slovenian CBI-24 and CBI-24-P demonstrated satisfactory psychometric properties and supported three-factor structures in the nurse and patient samples, providing contextually appropriate instruments for assessing caring behaviors in Slovenian EDs. Nurses and patients generally report high levels of caring behaviors, particularly in relation to professional knowledge, technical competence, confidentiality, and patient safety. Patients nevertheless rated several relational and responsiveness-related behaviors lower, including communication, proactive contact, concern, and time availability. Although some differences reached statistical significance, the corresponding effect sizes were small and therefore of limited practical importance.

In the patient sample, longer time to initial physician assessment and longer overall treatment duration were independently associated with lower total CBI-24-P scores after adjustment for sex, age, and mode of arrival. These associations should not be interpreted causally, as the cross-sectional design does not establish that longer waiting or treatment times directly reduced perceived caring. The model explained only a limited proportion of the variance, suggesting that additional clinical, interpersonal, and organizational factors are also relevant.

These findings support attention to both technical and relational dimensions of emergency nursing care. Strategies involving clear communication, regular updates during waiting, responsiveness to patients’ needs, and appropriate workflow and staffing organization may be relevant to patient-centered care. Prospective and intervention studies are needed to determine whether changes in these factors lead to improvements in perceived caring behaviors.

## Figures and Tables

**Table 1:** Sociodemographic characteristics of the nurses (N = 354) in Emergency Departments (EDs).

Sociodemographic Factors	N (%)
**Age (M ± SD)**	36.80 ± 10.19
**Range**	21–63 years
**Years of experience in the ED**	9.24 ± 9.17
**Total years of work experience**	14.59 ± 10.76
**Gender**	
Male	76 (21.5%)
Female	278 (78.5%)
**Age**	
21–30	126 (35.6%)
31–40	115 (32.5%)
41–50	69 (19.5%)
51–60	40 (11.3%)
61–70	4 (1.1%)
**Highest level of education**	
Secondary school	79 (22.3%)
Bachelor’s degree	236 (66.7%)
Master’s degree	38 (10.7%)
PhD	1 (0.3%)
**Total years of experience**	
≤5	82 (23.2%)
6–10	83 (23.4%)
11–15	53 (14.9%)
16–20	48 (13.6%)
>21	88 (24.9%)
**Years of experience in the ED**	
≤5	169 (47.7%)
6–10	92 (26.0%)
11–15	23 (6.5%)
16–20	28 (7.9%)
>21	42 (11.9%)
**Assessment of the quality of nursing care in ED**	
Excellent	90 (25.4%)
Good	165 (46.6%)
Satisfactory	77 (21.8%)
Neither good nor bad	17 (4.8%)
Poor	2 (0.6%)
Extremely poor	3 (0.8%)

N = number of respondents; M = mean; SD = standard deviation; % = percentage.

**Table 2:** Sociodemographic characteristics of the patients (N = 288).

Variable	N (%)
**Age (M ± SD)**	50.66 ± 19.40
**Range**	18–92
**Gender**	
Male	138 (47.9%)
Female	150 (52.1%)
**Age**	
18–30	55 (19.1%)
31–40	36 (12.5%)
41–50	57 (19.8%)
51–60	49 (17.0%)
61–70	32 (11.1%)
>70	59 (20.5%)
**ED mode of arrival**	
By ambulance	66 (22.9%)
With family members	130 (45.1%)
Independently	85 (29.5%)
From another ward	7 (2.4%)
**Duration of the overall treatment**	
<1 h	16 (5.6%)
1–2 h	43 (14.9%)
2–3 h	62 (21.5%)
3–4 h	67 (23.3%)
>4 h	100 (34.7%)
**Time to initial physician assessment**	
<30 min	99 (34.4%)
30–60 min	93 (32.3%)
60–90 min	36 (12.5%)
90–120 min	20 (6.9%)
>120 min	40 (13.9%)
**Recommendation of the visited ED to family and friends**	
Absolutely not	4 (1.4%)
Not sure	13 (4.5%)
Maybe	80 (27.8%)
Absolutely	191 (66.3%)

N = number of respondents; M = mean; SD = standard deviation; % = percentage.

**Table 3:** Assessment of perceived caring behaviors among patients and nurses in the ED.

Item	M (SD)		
	Nurses (N = 354)	Patients (N = 288)	Mean Difference
Attentively listening to the patient.	5.37 (0.81)	5.27 (1.00)	+0.10
2. Giving instructions or teaching the patient.	5.40 (0.88)	5.21 (0.94)	+0.19
3. Treating patients as individuals.	5.46 (0.81)	5.17 (1.15)	+0.29
4. Spending time with the patient.	4.84 (1.10)	4.84 (1.28)	0.00
5. Supporting the patient.	5.18 (0.92)	5.13 (1.10)	+0.05
6. Being empathetic or identifying with the patient.	5.32 (0.88)	5.05 (1.21)	+0.27
7. Helping the patient grow.	4.53 (1.24)	5.12 (1.09)	−0.59
8. Being patient or tireless with the patient.	5.06 (1.03)	5.10 (1.15)	−0.04
9. Knowing how to give shots, IVs, etc.	5.59 (0.85)	5.42 (0.85)	+0.17
10. Being confident with the patient.	5.47 (0.80)	5.29 (0.85)	+0.18
11. Demonstrating professional knowledge and skill.	5.42 (0.80)	5.43 (0.87)	−0.01
12. Managing equipment skillfully.	5.40 (0.76)	5.49 (0.76)	−0.09
13. Allowing the patient to express feelings about their disease and treatment.	5.16 (0.94)	5.14 (1.14)	+0.02
14. Including the patient in planning their care.	4.91 (1.03)	4.83 (1.35)	+0.08
15. Treating patient information confidentially.	5.69 (0.72)	5.53 (0.83)	+0.16
16. Returning to the patient voluntarily.	5.18 (0.99)	4.81 (1.44)	+0.37
17. Talking to the patient.	5.35 (0.92)	4.87 (1.48)	+0.48
18. Encouraging the patient to call if there are problems.	5.42 (0.93)	5.03 (1.37)	+0.39
19. Meeting the patient’s stated and unstated needs.	5.05 (0.95)	4.94 (1.33)	+0.11
20. Responding quickly to the patient’s call.	5.18 (0.92)	5.00 (1.37)	+0.18
21. Helping to reduce the patient’s pain.	5.54 (0.81)	5.31 (1.06)	+0.23
22. Showing concern for the patient.	5.45 (0.85)	5.06 (1.26)	+0.39
23. Giving the patient’s treatments and medications on time.	5.47 (0.85)	5.29 (1.02)	+0.18
24. Relieving the patient’s symptoms.	5.44 (0.88)	5.26 (1.08)	+0.18

N = number of respondents; M = mean; SD = standard deviation.

**Table 4:** Differences in CBI-24 domain scores according to demographic characteristics of nurses.

Variable	N	Knowledge and Skills	Relationally Supportive and Empathic Caring Behaviors	Assurance of Human Presence	Total
		M ± SD	M ± SD	M ± SD	M ± SD
**Gender**					
Male	76	5.33 ± 0.77	4.81 ± 0.77	4.94 ± 0.73	5.08 ± 0.67
Female	278	5.53 ± 0.62	5.11 ± 0.81	5.24 ± 0.75	5.34 ± 0.65
**Highest level of education**					
Secondary	79	5.48 ± 0.59	5.13 ± 0.74	5.27 ± 0.67	5.33 ± 0.58
BSc	236	5.53 ± 0.57	5.06 ± 0.76	5.17 ± 0.73	5.30 ± 0.60
Postgraduate (MSc, PhD)	39	5.24 ± 1.14	4.80 ± 1.15	5.06 ± 1.03	5.08 ± 1.05
**Age**					
21–30	126	5.44 ± 0.66	4.85 ± 0.85	5.06 ± 0.77	5.18 ± 0.66
31–40	115	5.48 ± 0.74	5.12 ± 0.83	5.18 ± 0.79	5.30 ± 0.73
41–50	69	5.49 ± 0.66	5.18 ± 0.72	5.30 ± 0.68	5.36 ± 0.62
51–60	40	5.66 ± 0.38	5.20 ± 0.70	5.35 ± 0.72	5.46 ± 0.49
61–70	4	5.52 ± 0.33	5.13 ± 0.63	5.21 ± 0.47	5.33 ± 0.43
**Total years of experience**					
≤5	82	5.42 ± 0.50	4.86 ± 0.84	5.08 ± 0.72	5.18 ± 0.57
6–10	83	5.48 ± 0.81	5.08 ± 0.85	5.13 ± 0.83	5.28 ± 0.77
11–15	53	5.58 ± 0.48	5.16 ± 0.58	5.33 ± 0.57	5.40 ± 0.47
16–20	48	5.46 ± 0.86	5.02 ± 0.99	5.16 ± 0.87	5.27 ± 0.84
>21	88	5.50 ± 0.61	5.13 ± 0.73	5.23 ± 0.74	5.33 ± 0.61
**Years of experience in the ED**					
≤5	169	5.48 ± 0.57	4.99 ± 0.78	5.13 ± 0.73	5.26 ± 0.60
6–10	92	5.50 ± 0.71	5.14 ± 0.82	5.21 ± 0.76	5.32 ± 0.70
11–15	23	5.39 ± 0.83	4.98 ± 0.84	5.22 ± 0.81	5.24 ± 0.79
16–20	28	5.37 ± 0.95	4.93 ± 1.01	5.07 ± 0.96	5.17 ± 0.90
>21	42	5.61 ± 0.55	5.19 ± 0.73	5.34 ± 0.67	5.43 ± 0.56

N = number of respondents; SD = standard deviation.

**Table 5:** Inferential analysis of differences in CBI-24 domain scores according to nurses’ demographic and professional characteristics.

Variable	Knowledge and Skills		Relationally Supportive and Empathic Caring Behaviors		Assurance of Human Presence		Total	
	U/χ^2^	r/η^2^H	U/χ^2^	r/η^2^H	U/χ^2^	r/η^2^H	U/χ^2^	r/η^2^H
Gender	8946.00 *	0.11	7817.00 **	0.19	7685.50 **	0.20	7849.00 **	0.18
Highest level of education	0.170	0.005	1.387	0.001	0.833	0.003	0.268	0.001
Age	5.607	0.005	11.560 *	0.022	9.363	0.015	8.736	0.014
Total years of experience	7.976	0.011	5.359	0.004	4.272	0.001	6.535	0.007
Years of experience in the ED	4.867	0.002	4.917	0.003	4.214	0.001	4.838	0.002

U = Mann–Whitney U statistic; χ^2^ = Kruskal–Wallis; *p* = statistical significance; ** = *p* < 0.01; * = *p* < 0.05, r/η^2^H = effect size.

**Table 6:** Differences in CBI-24-P domain scores according to demographic characteristics of patients.

Variable	N	Knowledge and Skills	Relationally Supportive and Empathic Caring Behaviors	Assurance of Human Presence	Total
		M ± SD	M ± SD	M ± SD	M ± SD
**Gender**					
Male	138	5.48 ± 0.63	5.22 ± 0.83	5.21 ± 0.92	5.27 ± 0.77
Female	150	5.39 ± 0.69	4.96 ± 1.05	4.92 ± 1.20	5.03 ± 0.96
**ED mode of arrival**					
Ambulance	66	5.24 ± 0.75	4.92 ± 0.96	5.01 ± 1.01	5.02 ± 0.89
Family members	130	5.47 ± 0.66	5.11 ± 0.97	5.05 ± 1.14	5.16 ± 0.89
Independently	85	5.48 ± 0.58	5.10 ± 0.93	5.06 ± 1.08	5.16 ± 0.84
Another ward	7	5.89 ± 0.16	5.89 ± 0.20	5.73 ± 0.48	5.83 ± 0.26
**Age**					
18–30	55	5.40 ± 0.66	4.92 ± 0.93	4.77 ± 1.05	4.97 ± 0.84
31–40	36	5.42 ± 0.72	5.18 ± 0.81	5.42 ± 0.84	5.24 ± 0.74
41–50	57	5.52 ± 0.52	5.02 ± 1.12	4.95 ± 1.42	5.10 ± 1.04
51–60	49	5.35 ± 0.69	5.01 ± 1.05	5.00 ± 1.10	5.08 ± 0.96
61–70	32	5.50 ± 0.76	5.36 ± 0.78	5.34 ± 0.84	5.38 ± 0.74
>70	59	5.40 ± 0.67	5.15 ± 0.89	5.22 ± 0.93	5.23 ± 0.81
**Time to initial physician assessment**					
<30 min	99	5.60 ± 0.57	5.34 ± 0.89	5.32 ± 1.00	5.39 ± 0.82
30–60 min	93	5.48 ± 0.59	5.12 ± 0.85	5.11 ± 0.94	5.19 ± 0.77
60–90 min	36	5.21 ± 0.56	4.85 ± 0.94	4.93 ± 1.00	4.95 ± 0.82
90–120 min	20	5.58 ± 0.53	5.39 ± 0.63	5.27 ± 0.71	5.38 ± 0.61
>120 min	40	5.02 ± 0.91	4.41 ± 1.13	4.30 ± 1.43	4.50 ± 1.09
**Duration of the overall treatment**					
<1 h	16	5.48 ± 0.53	5.31 ± 0.80	5.50 ± 0.52	5.42 ± 0.60
1–2 h	43	5.70 ± 0.53	5.38 ± 0.67	5.42 ± 0.65	5.46 ± 0.59
2–3 h	62	5.49 ± 0.66	5.30 ± 0.85	5.10 ± 1.12	5.26 ± 0.85
3–4 h	67	5.37 ± 0.60	4.90 ± 1.04	4.97 ± 1.13	5.02 ± 0.92
>4 h	100	5.31 ± 0.73	4.91 ± 1.03	4.87 ± 1.19	4.98 ± 0.96

N = number of respondents; SD = standard deviation.

**Table 7:** Inferential analysis of differences in CBI-24-P domain scores according to patients’ demographic and clinical characteristics.

Variable	Knowledge and Skills		Relationally Supportive and Empathic Caring Behaviors		Assurance of Human Presence		Total	
	U/χ^2^	r/η^2^H	U/χ^2^	r/η^2^H	U/χ^2^	r/η^2^H	U/χ^2^	r/η^2^H
Gender	9934.00	0.04	9005.00	0.11	9352.50	0.08	9144.50	0.10
ED mode of arrival	8.749 *	0.022	10.803 *	0.031	3.625	0.002	7.898 *	0.023
Age	2.483	<0.001	6.472	0.005	10.039	0.018	7.129	0.008
Time to initial physician assessment	27.672 **	0.084	34.143 **	0.107	24.734 **	0.073	32.506 **	0.101
Duration of the overall treatment	16.242 **	0.043	14.327 **	0.036	8.414	0.016	13.150 *	0.032

U = Mann–Whitney U statistic; χ^2^ = Kruskal–Wallis; *p* = statistical significance; ** = *p* < 0.01; * = *p* < 0.05, r/η^2^H = effect size.

**Table 8:** Correlations between caring-behaviors scores and age, nurses’ total work experience, nurses’ ED experience, time to initial physician assessment, and overall treatment duration.

Three Domains	Age ^1^	Total Years of Experience ^1^	Years of Experience in ED ^1^	Age ^2^	Time to Initial Assessment ^2^	Duration of Treatment ^2^
Knowledge and skills	0.129 *	0.108 *	0.107 *	0.032	−0.260 **	−0.191 **
Relationally supportive and empathic caring behaviors	0.140 **	0.104 *	0.072	0.111 **	−0.293 **	−0.194 **
Assurance of human presence	0.151 *	0.099	0.094	0.161 **	−0.270 **	−0.163 **
Total	0.147 *	0.107 *	0.097	0.131 **	−0.299 **	−0.195 **

^1^ = statistic results for nurse sample; ^2^ = statistic results for patient sample; * *p* < 0.05; ** *p* < 0.01.

**Table 9:** Multiple linear regression analysis of factors associated with the total CBI-24-P score among patients.

Model		B	SE	β	t	*p*-Value	95% Confidence Interval for B	
							Lower	Upper
1	Intercept (CBI-24-P)	5.775	0.292		19.763	<0.001	5.200	6.350
	Gender	−0.215	0.099	−0.122	−2.180	0.030	−0.409	−0.021
	Age	0.065	0.029	0.131	2.279	0.023	0.009	0.122
	Time to initial assessment	−0.145	0.038	−0.226	−3.845	<0.001	−0.219	−0.071
	Duration of treatment	−0.105	0.042	−0.149	−2.505	0.013	−0.188	−0.023
	ED mode of arrival	0.092	0.064	0.081	1.426	0.155	−0.035	0.218

R = 0.363; R^2^ = 0.132; Adjusted R^2^ = 0.116; SEE = 0.828, Durbin–Watson = 1.612; F(5282) = 8.55; *p* < 0.001.

**Table 10:** Multiple linear regression analysis of factors associated with the total CBI-24 score among nurses.

Model		B	SE	β	t	*p*-Value	95% Confidence Interval for B	
							Lower	Upper
1	Intercept (CBI-24)	110.512	5.203		21.243	<0.001	100.287	120.737
	Age	0.198	0.149	0.127	1.329	0.185	−0.095	0.491
	Gender	6.845	1.912	0.178	3.580	<0.001	3.086	10.604
	Total years of experience in ED	−0.312	0.089	−0.212	−3.506	<0.001	−0.487	−0.137

R = 0.489; R^2^ = 0.239; Adjusted R^2^ = 0.233; SEE = 0.867; Durbin–Watson = 1.943; F(3350) = 36.72; *p* < 0.001.

## Data Availability

The original contributions of this study are included in the article; further inquiries can be directed to the corresponding author.

## Abbreviations

The following abbreviations are used in this manuscript:

AGFI	Adjusted Goodness-of-Fit Index
α	Cronbach’s Alpha
CBI-24	Caring Behaviors Inventory-24
CBI-24-P	Caring Behaviors Inventory-24, patient version
CFA	Confirmatory Factor Analysis
CFI	Comparative Fit Index
χ^2^	Chi-Square Statistic
CMIN/DF	Chi-Square Minimum divided by Degrees of Freedom
CVI	Content Validity Index
ΔM	Mean Difference
df	Degrees of Freedom
ED	Emergency Department
η^2^H	Ordinal Eta-Squared effect size for the Kruskal–Wallis Test
GFI	Goodness-of-Fit Index
H	Kruskal–Wallis H Statistic
I-CVI	Item-level Content Validity Index
IFI	Incremental Fit Index
κ*	Modified Kappa Statistic
KMO	Kaiser–Meyer–Olkin Index
M	Mean
Max/min	Maximum/minimum
N	Sample size
NFI	Normed Fit Index
ω	McDonald’s Omega Reliability Coefficient
PCA	Principal Component Analysis
R	Rosenthal’s effect size
RFI	Relative Fit Index
RMSEA	Root Mean Square Error of Approximation
r_s_	Spearman’s Rank Correlation Coefficient
S-CVI	Scale-level Content Validity
S-CVI/Ave	Average Scale Validity Index
S-CVI/UA	Scale-level Content Validity Index based on Universal Agreement
SD	Standard Deviation
U	Mann–Whitney U Statistics

## Appendix A. Principal Component Analysis (PCA)

**Table A1:** Rotated Component Matrix (PCA) with Total Variance Explained and Reliability Statistics for CBI-24-P.

	Subscale		
Item	1 Assurance of Human Presence	2 Relationally Supportive and Empathic Caring Behaviors	3 Knowledge and Skills
CBI-24-P-17	0.786	0.421	-
CBI-24-P-23	0.751	-	0.430
CBI-24-P-24	0.746	-	0.409
CBI-24-P-20	0.742	-	-
CBI-24-P-22	0.728	0.432	-
CBI-24-P-18	0.711	0.403	-
CBI-24-P-19	0.702	0.516	-
CBI-24-P-21	0.622	-	0.499
CBI-24-P-16	0.594	0.482	-
CBI-24-P-2	-	0.782	-
CBI-24-P-1	-	0.751	-
CBI-24-P-7	-	0.702	-
CBI-24-P-6	0.475	0.691	-
CBI-24-P-3	0.510	0.690	-
CBI-24-P-5	0.407	0.675	-
CBI-24-P-4	0.523	0.646	-
CBI-24-P-14	-	0.626	-
CBI-24-P-13	-	0.590	-
CBI-24-P-8	0.506	0.557	-
CBI-24-P-12	-	-	0.757
CBI-24-P-10	-	-	0.720
CBI-24-P-9	-	-	0.681
CBI-24-P-15	-	-	0.637
CBI-24-P-11	0.440	-	0.607
Total variance explained [%]	58.960	5.924	5.111
Cronbach’s α	0.945	0.946	0.849
McDonald’s ω (ML)	0.948	0.948	0.851

Varimax rotation without Kaiser normalization was applied. Rotation converged in 8 iterations.

**Table A2:** Total Variance Explained for Components (PCA) of the CBI-24-P.

	Initial Eigenvalues			Extraction Sums of Squared Loadings			Rotation Sums of Squared Loadings		
	Eigenvalue	% Variance	Cumulative %	Eigenvalue	% Variance	Cumulative %	Eigenvalue	% Variance	Cumulative %
1	14.150	58.960	58.960	14.150	58.960	58.960	6.591	27.464	27.464
2	1.422	5.924	64.884	1.422	5.924	64.884	6.263	26.094	53.557
3	1.227	5.111	69.995	1.227	5.111	69.995	3.945	16.438	69.995
4	0.890	3.708	73.704						
5	0.669	2.787	76.491						
6	0.608	2.534	79.025						
7	0.545	2.271	81.296						
8	0.478	1.994	83.290						
9	0.439	1.828	85.118						
10	0.423	1.764	86.881						
11	0.358	1.492	88.373						
12	0.354	1.475	89.849						
13	0.332	1.385	91.233						
14	0.285	1.189	92.422						
15	0.263	1.095	93.517						
16	0.248	1.033	94.550						
17	0.217	0.903	95.454						
18	0.206	0.859	96.312						
19	0.185	0.771	97.084						
20	0.171	0.711	97.794						
21	0.159	0.664	98.459						
22	0.146	0.609	99.068						
23	0.119	0.494	99.562						
24	0.105	0.438	100.000						

**Table A3:** Rotated Component Matrix (PCA) with Total Variance Explained and Reliability Statistics for CBI-24.

	Subscale		
Item	1 Knowledge and Skills	2 Relationally Supportive and Empathic Caring Behaviors	3 Assurance of Human Presence
CBI-24-10	0.839	-	-
CBI-24-9	0.829	-	-
CBI-24-11	0.756	-	-
CBI-24-21	0.721	-	0.404
CBI-24-23	0.705	-	0.428
CBI-24-15	0.679	-	-
CBI-24-12	0.669	-	-
CBI-24-24	0.651	-	0.423
CBI-24-3	0.625	0.484	-
CBI-24-2	0.577	0.514	-
CBI-24-22	0.568	-	0.513
CBI-24-5	-	0.773	-
CBI-24-7	-	0.762	-
CBI-24-4	-	0.744	-
CBI-24-6	-	0.684	-
CBI-24-8	-	0.626	-
CBI-24-1	0.482	0.592	-
CBI-24-19	-	-	0.744
CBI-24-16	-	-	0.718
CBI-24-18	0.402	-	0.661
CBI-24-17	-	-	0.656
CBI-24-20	-	-	0.608
CBI-24-14	-	0.548	0.551
CBI-24-13	-	0.499	0.507
Total variance explained [%]	54.272	7.808	4.546
Cronbach’s α	0.945	0.890	0.901
McDonald’s ω (ML)	0.946	0.892	0.901

**Table A4:** Total Variance Explained for Components (PCA) of the CBI-24.

	Initial Eigenvalues			Extraction Sums of Squared Loadings			Rotation Sums of Squared Loadings		
	Eigenvalue	% Variance	Cumulative %	Eigenvalue	% Variance	Cumulative %	Eigenvalue	% Variance	Cumulative %
1	13.025	54.272	54.272	13.025	54.272	54.272	6.602	27.508	27.508
2	1.874	7.808	62.080	1.874	7.808	62.080	4.963	20.681	48.188
3	1.091	4.546	66.626	1.091	4.546	66.626	4.425	18.438	66.626
4	0.761	3.171	69.797						
5	0.745	3.104	72.901						
6	0.679	2.828	75.729						
7	0.591	2.465	78.193						
8	0.543	2.262	80.456						
9	0.491	2.046	82.502						
10	0.466	1.943	84.445						
11	0.432	1.800	86.244						
12	0.378	1.576	87.821						
13	0.372	1.549	89.370						
14	0.337	1.404	90.773						
15	0.311	1.297	92.070						
16	0.293	1.220	93.291						
17	0.260	1.085	94.375						
18	0.248	1.033	95.408						
19	0.227	0.947	96.355						
20	0.210	0.875	97.230						
21	0.199	0.827	98.058						
22	0.168	0.698	98.756						
23	0.158	0.660	99.416						
24	0.140	0.584	100.000						

**Table A5:** Content Validity Index and modified Kappa coefficient for CBI-24.

Item	N	S	I-CVI	P_C_	*κ**	Evaluation
Attentively listening to the patient.	10	10	1	0.00098	1	Excellent
Giving instructions or teaching the patient.	10	10	1	0.00098	1	Excellent
Treating patient as an individual.	10	10	1	0.00098	1	Excellent
Spending time with patient.	10	10	1	0.00098	1	Excellent
Supporting the patient.	10	10	1	0.00098	1	Excellent
Being empathetic or identifying with the patient.	10	10	1	0.00098	1	Excellent
Helping the patient grow.	10	10	1	0.00098	1	Excellent
Being patient or tireless with the patient.	10	10	1	0.00098	1	Excellent
Knowing how to give shots, IVs, etc.	10	10	1	0.00098	1	Excellent
Being confident with the patient.	10	10	1	0.00098	1	Excellent
Demonstrating professional knowledge and skill.	10	10	1	0.00098	1	Excellent
Managing equipment skillfully.	10	10	1	0.00098	1	Excellent
Allowing the patient to express feelings about his or her disease and treatment.	10	10	1	0.00098	1	Excellent
Including the patient in planning his or her care.	10	10	1	0.00098	1	Excellent
Treating patient information confidentially.	10	10	1	0.00098	1	Excellent
Returning to the patient voluntarily.	10	10	1	0.00098	1	Excellent
Talking to the patient.	10	10	1	0.00098	1	Excellent
Encouraging the patient to call if there are problems.	10	10	1	0.00098	1	Excellent
Meeting the patient’s stated and unstated needs.	10	10	1	0.00098	1	Excellent
Responding quickly to the patient’s call.	10	10	1	0.00098	1	Excellent
Helping to reduce the patient’s pain.	10	10	1	0.00098	1	Excellent
Showing concern for the patient.	10	10	1	0.00098	1	Excellent
Giving the patient’s treatments and medications on time.	10	10	1	0.00098	1	Excellent
Relieving the patient’s symptoms.	10	10	1	0.00098	1	Excellent

N = total number of experts; S = number of experts who rated the item as relevant; I-CVI = item-level content validity index; $P_{c}$ = probability of chance agreement; $\kappa^{*}$ = modified kappa coefficient. Evaluation of $\kappa^{*}$: fair = 0.40–0.59, good = 0.60–0.74, and excellent ≥ 0.75.

**Table A6:** Content Validity Index and modified Kappa coefficient for CBI-24-P.

Item	N	S	I-CVI	P_C_	*κ**	Evaluation
The nurse listened to me as a patient.	10	10	1	0.00098	1	Excellent
The nurse gave me instructions or teaching.	10	10	1	0.00098	1	Excellent
The nurse treated me as an individual.	10	10	1	0.00098	1	Excellent
The nurse spent sufficient time with me as a patient.	10	10	1	0.00098	1	Excellent
The nurse supported me as a patient.	10	10	1	0.00098	1	Excellent
The nurse was being empathetic or identified with me as a patient.	10	10	1	0.00098	1	Excellent
The nurse helped me to grow.	10	9	0.9	0.00977	0.899	Excellent
The nurse was being patient or tireless with me as a patient.	10	10	1	0.00098	1	Excellent
The nurse knew how to give shots, IVs, etc.	10	9	0.9	0.00977	0.899	Excellent
The nurse was confident with me as a patient.	10	10	1	0.00098	1	Excellent
The nurse demonstrated professional knowledge and skill.	10	9	0.9	0.00977	0.899	Excellent
The nurse managed equipment skillfully.	10	9	0.9	0.00977	0.899	Excellent
The nurse allowed me to express feelings about my disease and treatment.	10	10	1	0.00098	1	Excellent
The nurse included me as a patient in planning my care.	10	10	1	0.00098	1	Excellent
The nurse treated my personal information confidentially.	10	10	1	0.00098	1	Excellent
The nurse returned to check on me voluntarily.	10	10	1	0.00098	1	Excellent
The nurse talked to me during care.	10	10	1	0.00098	1	Excellent
The nurse encouraged me to call if there are problems.	10	10	1	0.00098	1	Excellent
The nurse met both my expressed and unexpressed needs.	10	10	1	0.00098	1	Excellent
The nurse responded quickly to my call.	10	10	1	0.00098	1	Excellent
The nurse helped to reduce pain.	10	10	1	0.00098	1	Excellent
The nurse showed concern for me as a patient.	10	10	1	0.00098	1	Excellent
The nurse administered my treatments and medications on time.	10	10	1	0.00098	1	Excellent
The nurse helped relieve my symptoms.	10	10	1	0.00098	1	Excellent

N = total number of experts; S = number of experts who rated the item as relevant; I-CVI = item-level content validity index; $P_{c}$ = probability of chance agreement; $\kappa^{*}$ = modified kappa coefficient. Evaluation of $\kappa^{*}$: fair = 0.40–0.59, good = 0.60–0.74, and excellent ≥ 0.75.

**Table A7:** Goodness-of-fit of different models (CBI-24).

Goodness-of-Fit Indices Acceptable Values	RMSEA [<0.10]	CMIN/DF [<3]	IFI [≥0.90]	RFI [≥0.90]	NFI [≥0.90]	GFI [≥0.90]	AGFI [≥0.90]	CFI [≥0.90]
CBI-24 (Original 4-factor model among nurses)	0.158	9.813	0.666	0.608	0.642	0.64	0.61	0.665
CBI-24 (Slovenian version for nurses)	0.072	2.83	0.914	0.872	0.891	0.889	0.864	0.912
CBI-24 (Original 4-factor model among patients)	0.162	8.531	0.705	0.617	0.678	0.69	0.63	0.702
CBI-24-P (Slovenian Version for patients)	0.038	1.41	0.992	0.906	0.97	0.94	0.91	0.970

RMSEA = Root Mean Square Error of Approximation; CMIN/DF = Chi-Square Minimum Divided by Degrees of Freedom; IFI = Incremental Fit Index; RFI = Relative Fit Index; NFI = Normed Fit Index; GFI = Goodness-of-Fit Index; AGFI = Adjusted Goodness-of-Fit Index; CFI = Comparative Fit Index.
